# Timing of tertiary trauma surveys during a time of increased trauma presentations – The Alice Springs Hospital Finke Desert Race experience

**DOI:** 10.1016/j.heliyon.2024.e31433

**Published:** 2024-05-18

**Authors:** Kirby Laslett, Chris Perry, Jayantha Senaratne, Charles Coventry

**Affiliations:** a Department of General Surgery, Alice Springs Hospital, 6 Gap Road, The Gap, Northern Territory, Australia, 0870; b Research Support Coordinator, Central Australia Health Region, NT Health, 6 Gap Road, The Gap, Northern Territory, Australia, 0870; c Department of Trauma Surgery, Royal Adelaide Hospital, Central Adelaide Local Health Network, Port Road, Adelaide, South Australia, Australia, 5000

**Keywords:** Trauma, Tertiary trauma survey, Rural surgery, General surgery, Motorsport

## Abstract

**Purpose:**

The Finke Desert Race is an offroad motorbike and buggy race held annually in central Australia. Owing to the treacherous conditions, this race sees a significant influx of trauma presentations to Alice Springs Hospital, the closest rural hospital. Completion of a tertiary trauma survey (TTS) within 24 hours of a patient's admission is part of standard trauma management.

**Method:**

A retrospective analysis was undertaken of trauma presentations managed by general surgery over a 5-day period of the Finke Desert Race weekend, compared to a 3-month control period from February to April of the same year. To be included, patients met the criteria for completion of a TTS.

**Results:**

The total number of trauma presentations over the 5-day period of the race weekend was 18 (an incidence rate of 3.6 cases/day), compared to a total of 31 in the 3-month control period (an incidence rate of 0.36 cases/day). The daily rate of major trauma presentations during the Finke race weekend was 9.9 times greater than during the control period. Completion of TTS was missed in only 5.6 % of patients over the Finke weekend, compared to 14.3 % of patients in the control period. The median time from presentation to the emergency department to completion of TTS during the Finke weekend was 20 h 19 min, compared to 20 h 36 min during the control period.

**Conclusion:**

Despite the substantial influx of trauma during the race weekend, fewer patients missed having a TTS completed compared to the control period. The median time taken to completion of TTS was similar between the two time periods. These findings suggest that the general surgery department was able to maintain standard trauma management principles.

## Highlights

• The Finke Desert Race is an offroad motorbike race held annually in central Australia.
• This race sees an almost 10-fold increase in the rate of trauma presentations to Alice Springs Hospital.
• Despite this, the general surgery department was able to maintain standard trauma management principles.
• Fewer patients missed having a tertiary trauma survey completed compared to the control period.
• The median time taken to completion of tertiary trauma survey was similar.

## 1 Introduction

Every year over the June long weekend, the Finke Desert Race is held in central Australia. The race is a ‘there and back’ race, from Alice Springs to Finke (also known as Aputula Community) and back. As the name suggests, this is an offroad race through the desert, on rough terrain comprised of mostly sand dunes and dirt roads [1]. Competitors of the race compete in either the motorbike or buggy categories, with the majority of participants competing on motorbikes. The race distance is approximately 226 km each way, total distance approximately 452 km, with multiple makeshift checkpoints for fuel and pit stops along the way [1]. The Finke Desert Race is one of the most challenging off-road races in the world and is known as “Australia's greatest desert race” [1]. Competitors come from all around Australia to participate in or watch the event as spectators. It is estimated that the population of the Alice Springs area over the course of the Finke weekend increases by a further 12,000 people, adding to the usual population of approximately 25,000 people [1,2].

The Finke Desert Race presents a number of challenges to health care providers, as it has sections of track in remote and difficult-to-access areas [3]. The closest hospital to the racetrack is Alice Springs Hospital (ASH), a 210-bed hospital with 4 operating theatres, CT and MRI scanners, a 10 bed ICU/HDU, and general and orthopaedic surgical departments [3]. The general surgery department is called upon to manage patients who present with significant trauma, from their arrival in the emergency department in the instance of severe trauma warranting a trauma call; coordinating transfers of trauma patients to tertiary trauma centres; and after admission to the ward from the emergency department. Major trauma patients requiring tertiary-level care are transferred out from ASH to the Royal Adelaide Hospital (RAH), the closest trauma centre (some 1,500 km away, translating into a 2–4 hour flight). Aeromedical retrieval of patients from ASH to the RAH is provided by the Medical Retrieval and Consultation Centre (MRaCC), which is staffed by Royal Flying Doctor Service (RFDS) fixed-wing aircraft. Depending on the injuries sustained, trauma patients are managed in conjunction with the orthopaedic surgery, ICU, anaesthetic pain service, medical and other subspecialty teams where needed.

ASH does not have a trauma team, yet every year the hospital is called upon to cope with a significant increase in the influx of trauma presentations to the emergency department over a period of a few days. The resources available are significantly limited. The surgical, theatre, radiographer, and emergency department teams increase staffing over the course of the weekend in anticipation of the event, however other more finite resources required to manage trauma such as the number of beds in ICU and on the wards remain the same. The increase in trauma presentations to ASH is in addition to the existing workload of non-trauma related admissions, which is also often increased over the course of Finke due to the increase in the number of visitors to the area for the race.

This study aims to describe the increase in trauma presentations during this major motorsport event and to assess the quality of trauma care provided by the general surgical team during this time.

## 2 Methods

### 2.1 Study design

This retrospective cohort study was conducted at a single centre. The Central Australian Human Research Ethics Committee granted ethical approval prior to commencement of this study (CAHREC number 23–4641). Reporting was performed based on the recommendations by Strengthening the Reporting of Observational Studies in Epidemiology (STROBE) [4].

A retrospective analysis was performed of major trauma presentations managed by general surgery over a 5-day period of the Finke Desert Race weekend in June 2023, compared to a 3-month control period from February to April of the same year. Data was collected and analysed over June–July 2023.

### 2.2 Setting and comparison

The study was conducted at Alice Springs Hospital ASH), a publicly funded rural hospital in the Northern Territory of Australia. ASH does not have a dedicated trauma service, the initial trauma management is primarily led by the general surgery department, often in conjunction with other subspecialty teams. The racetrack for the Finke Desert Race stretches from within Alice Springs town to the remote community of Finke 226 km away [1]. Injured competitors or spectators may need to be evacuated from along any stretch of this track, which are mostly comprised of dirt or sandy roads where there are tracks available. The inaccessibility and remoteness of parts of the track mean that retrieval to ASH may take up to several hours.

The comparison time period of February to April 2023 was chosen as a time period that would capture the average workload of the general surgery department at most times of the year, avoiding the hotter months of the peak of summer, and ensuring no crossover with the Finke race period or the immediate lead up to the race, as during the month of May there is an increase in the number of competitors doing practice runs on the racetrack in preparation for the race weekend.

### 2.3 Patient characteristics and outcomes measured

Both electronic and paper records were analysed, this took the form of individual paper patient files, radiology reports, admission data from the emergency department, patient lists from the relevant time periods, and data collected prospectively by the general surgery department for the purposes of weekly audit meetings.

Patient demographics and details of the presentation to the emergency department were collected; including age, gender, date and time of presentation, trauma call status, time taken to review by general surgery, injuries sustained, the injury severity score (ISS), and whether the injury was sustained at the Finke Desert Race or in relation to preparations for the Finke Desert Race (ie, injuries sustained during practice for the race in the control period).

The primary outcome of interest was rate of missed TTS and the average time taken to completion of TTS between the two groups. Secondary outcomes of interest included the time taken to general surgical review, need for operative intervention, need for transfer to a tertiary centre and mortality.

Best practice guidelines in the management of trauma patients dictates that patients should undergo a tertiary trauma survey (TTS), ideally within 24 h of the incident [5]. A TTS involves a thorough head-to-toe examination of the patient, at a time after the initial shock of the insult has worn off, and when patients are able to be assessed for the extent of injuries more accurately, after the more obvious and life-threatening injuries have been addressed [5,6]. The significant influx of trauma patients to a small general surgical department without a trauma team could mean that more time consuming, although important, aspects of trauma care could be delayed or even missed. Analysing the proportion of completed TTS and the time taken for TTS to occur has been used as a surrogate indicator for how well the general surgical department is coping with the significant increase in workload over the Finke weekend. Additionally, assessment of the time taken from ED assessment of the patient until they are seen by the general surgery team adds to the overall picture of how well the general surgery department is coping with the increase in workload.

Inclusion criteria were: patients who presented to ASH with major trauma during the study periods (9–13th June 2023 and 1st February to April 30, 2023). Major trauma criteria included: major traumatic injury (injury severity score (ISS) > 12) or significant mechanism of injury (motor vehicle or motorbike accident >60 kmph, fall from >2 m, penetrating injury to thorax/abdomen/head). Patients of all ages were included. Exclusion criteria included patients who died within 24 h of presentation and patients who were discharged/self-discharged directly from the emergency department.

### 2.4 Statistical analysis

The data were analysed to determine the frequency (percentage) for categorical variables, which was then compared using Chi-Squared test. Continuous variables were analysed and displayed as median and interquartile ranges and compared using Mann-Whitney *U* test. All statistical analyses were performed using Stata 17 (2021. *Stata* StataCorp LLC). The threshold for statistical significance in this study was set at 95 %.

## 3 Results

### 3.1 Major trauma presentations

During the Finke race period, 18 patients met the inclusion criteria in 5 days (an incidence rate of 3.6 cases/day). In the 3-month control period, there were 31 patients who met the inclusion criteria over an 85-day period (an incidence rate of 0.36 cases/day). This equates to just over 1 trauma presentation every 3 days.

An incident rate ratio (IRR) was computed comparing daily trauma presentations during the Finke race period (5 days) to the 3-month control period (85 days), in 2023. The IRR of daily trauma presentations between these two time periods was 9.9 (95%CI 5.2–18.2). This difference was highly statistically significant (p = 0.000). The daily rate of trauma presentations to Alice Springs Hospital during the 5-day Finke race period was 9.9 times greater than the daily trauma presentation rate in the 3-month control period.

### 3.2 Patient demographics

The average age of patients admitted with major trauma during the Finke period was 33, which was similar to the control period with an average age of 34. 100 % of the trauma patients admitted over the Finke weekend were male, compared to 64.5 % male in the control period.

### 3.3 Primary outcome

A tertiary trauma survey (TTS) was completed in 83.3 % (15/18) of all trauma presentations during the Finke race period and in 75 % (21/28) of all trauma presentations during the 3-month control period (3 of the 31 patients were transferred to tertiary hospitals in the control period and so were not eligible for TTS completion). The proportion of TTS completed during the Finke race period was 7.3 % higher than in the control period. This was not determined to be statistically significant (p = 0.5).

**Fig. 1:**
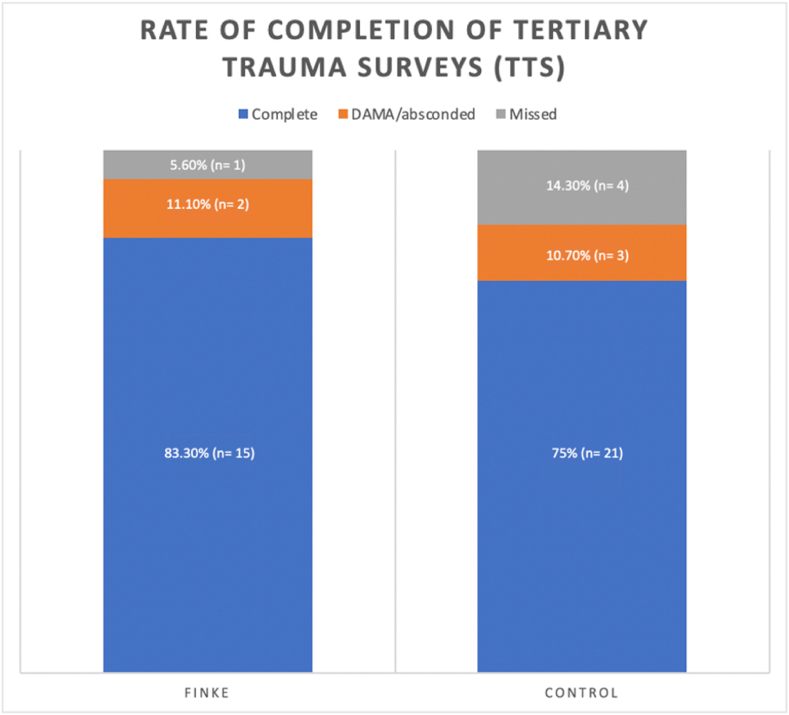
Graph showing breakdown of completed TTS, missed TTS and those incomplete due to patient discharging against medical advice (DAMA) or absconding prior to TTS completion.

Completion of TTS was missed in only one patient (5.6 %) over the Finke weekend, compared to four (14.3 %) patients in the control period. This result was not statistically significant (p = 0.666). 11.1 % (2/18) of patients during the Finke period and 10.7 % (3/28) of patients in the control period did not have a TTS completed as they discharged against medical advice (DAMA) or absconded from hospital prior to TTS completion (Fig. 1).

The median time from presentation to the emergency department to completion of TTS during the Finke weekend was 20 h 19 min (IQR 10 h 54 min–39 h 50 min), compared to 20 h 36 min (IQR 15 h 40 min–24 h 33 min) during the control period, this was not statistically significant (p = 0.78).

Of the TTS completed in each time period, new investigations were ordered as a result of the findings of the TTS in 13.3 % (2/15) of surveys completed during the Finke period, and 4.8 % (1/21) of surveys completed during the control period. Of these new investigations ordered, only 1 identified a new injury that was not previously identified during the patient's assessment.

### 3.4 Secondary outcomes

The median time from presentation to the emergency department to review by general surgery was 3 h, 55 min (234.5 min) for the Finke period, compared to 3 h, 38 min (218 min) for the control period. Distributions of the review times for the different time periods were similar, as assessed by visual inspection. There was no statistically significant difference in the general surgical review times (in minutes) between the two time periods (p = 0.56).

**Fig. 2:**
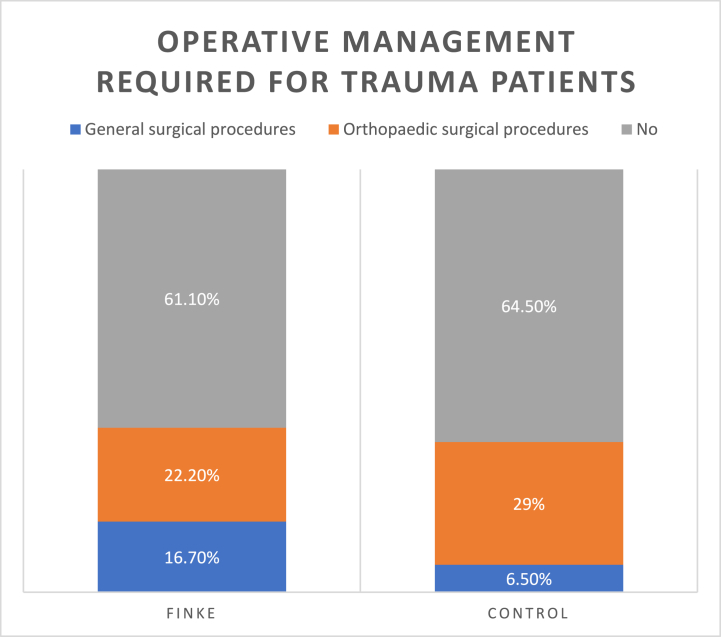
Graph showing breakdown of proportion of trauma patients that required operative management, and which specialty performed these operations.

During the Finke weekend, none of the patients admitted with trauma required transfer to a tertiary hospital for further management (0/18), whereas 9.7 % (3/31) of the patients admitted during the control period required transfer to a tertiary hospital. 38.9 % (7/18) of trauma patients admitted over the Finke weekend required operative management at Alice Springs Hospital, of these patients 16.7 % (3/18) required general surgical procedures, and 22.2 % (4/18) required orthopaedic surgical procedures. The proportion of patients requiring operative management over the control period was similar, 35.5 % (11/31) of trauma patients required surgery; 6.5 % (2/31) of patients required general surgical procedures, and 29 % (9/31) required orthopaedic procedures (Fig. 2).

In the 3-month control period from February to April there were 31 trauma presentations, and 12.9 % (4/31) were associated with preparation and practice for the Finke race. There were no mortalities in hospital in either the Finke race period or the control period.

## 4 Discussion

This study demonstrates that during the Finke desert race there was an almost 10-fold increase in the rate of trauma presentations per day, which in addition to the usual busy workload for the general surgery department puts substantial pressure on the department. Completion of TTS was chosen as a surrogate measure of ability to cope with this increase in workload as it forms an important, although time consuming, part of standard trauma management principles. Pleasingly, the proportion of completed TTS over the Finke race weekend was similar to the control period. Additionally, the time to completion of TTS was similar between the two groups, and also within 24 h of admission, which is consistent with standard trauma management guidelines [6].

This likely reflects the changes to staffing and patient flow put in place to manage increased demand on trauma services during the Finke race. Within the general surgical department, increased staffing for the duration of the race was arranged. Usually, the general surgery team is staffed by 1 on call registrar for the duration of the weekend, this was increased to 3 on call registrars for the race weekend (including Friday and Monday, as these were also race days). In addition to this, there was an additional resident or intern rostered on for the afternoon shift, when there is usually only 1 resident or intern working at this time. It is likely that this increased staffing contributed in a large part to the general surgery team's ability to keep up with the increased workload.

Other suggested reasons for why the team were able to manage the increase in trauma presentations may relate to increased trauma preparedness. In the lead up to the race weekend, staff at ASH arranged for trauma simulation sessions to be run in the emergency department, inviting team members from the emergency department, general surgery, orthopaedic surgery, and radiology to participate in simulation exercises. These simulations comprised a multidisciplinary team of doctors, nurses, allied health, and radiographers. It is possible that increased trauma preparedness, awareness, and familiarity over the course of the race weekend may have contributed to increased performance and management of major trauma patients. Being an observational study, causality cannot be inferred from these results, but merely a comment on possible explanations for these results.

One of the strengths of this audit is that the data was collected by a single author, which allowed for consistency in reporting and keeping to the inclusion and exclusion criteria. The authors were comprised of team members who were very familiar with the inner workings and running of the general surgery department, the conditions of the race weekend, the staffing and resources available, along with the challenges of not only the race weekend but the central Australian environment and health system as a whole.

Due to the use of paper notes at Alice Springs Hospital, there are some details that are possibly less accurate than if an electronic medical record system were used, particularly in the few instances where the exact time that the TTS was completed was not always recorded. In these instances, an approximate time was determined based on a combination of information available from the nursing notes and vital signs observation charts.

Another limitation is in the accurate representation of the time to be reviewed by general surgery. This time was often extended by an overnight wait, which reflects the fact that ASH does not staff a night surgical registrar, but rather the registrar is on call from home. This means that the registrar is not called after hours about non-time critical referrals, and these are reviewed at an early morning pre-round. Therefore, a delay in review is not always reflective of the response time or busyness of the registrar. This is consistent across both the Finke and control groups, and so there is a fair comparison between the two groups. In addition, this study did not capture the competing general surgical workload, which may also impact on TTS completion and ED review times.

Although every effort was made to capture data for all trauma patients seen by general surgery within the specified time periods, due to the nature of the record keeping for these trauma patients it is possible that some patients that were transferred to a tertiary centre during the control period were missed, particularly if they were transferred from the emergency department soon after their arrival.

The small sample size of this study limits the ability to interpret and extrapolate the findings, the underpowered nature of this study means that differences between the groups were not statistically significant. Future research in this area would benefit from a greater sample size.

Major motorsport events have the potential to increase demand on local health services. This study demonstrates that adequate preparation and utilisation of resources can prepare regional hospitals for such events and the influx of patients they may bring. Although preparatory measures will depend on the local context and specific situation, the measures put in place at our institution appear to result in adherence to trauma protocols despite an increase in demand.

## 5 Conclusion

Despite the substantial influx of trauma during the race weekend, fewer patients missed having a TTS completed prior to discharge compared to the control period. The median time taken to completion of TTS was similar between the two time periods. These findings suggest that the model used by the ASH general surgical team is appropriate to manage the increased number of trauma presentations during a major motorsport event. The authors hope to encourage trauma preparedness training and familiarisation with trauma management principles in the lead up to major motorsport events such as the Finke Desert Race, in addition to staffing appropriately to optimise patient safety.

## Funding

None.

## Data availability statement

Data will be made available upon request.

## Ethics approval

This study was reviewed and approved by the Central Australian Human Research Ethics Committee with the approval number: CAHREC 23–4641, dated June 26, 2023, granted prior to commencement of this study. The ethics committee granted a waiver of consent from individual participants.

## CRediT authorship contribution statement

**Kirby Laslett:** Writing – review & editing, Writing – original draft, Visualization, Resources, Project administration, Methodology, Investigation, Formal analysis, Data curation, Conceptualization. **Chris Perry:** Writing – review & editing, Software, Formal analysis, Data curation. **Jayantha Senaratne:** Supervision, Conceptualization. **Charles Coventry:** Writing – review & editing, Supervision, Project administration, Methodology, Data curation, Conceptualization.

## Declaration of competing interest

The authors have no conflicts of interest to declare.
